# Touch-Responsive Hydrogel for Biomimetic Flytrap-Like Soft Actuator

**DOI:** 10.1007/s40820-022-00931-4

**Published:** 2022-09-05

**Authors:** Junjie Wei, Rui Li, Long Li, Wenqin Wang, Tao Chen

**Affiliations:** 1 Key Laboratory of Marine Materials and Related Technologies, Zhejiang Key Laboratory of Marine Materials and Protective Technologies, Ningbo Institute of Materials Technology and Engineering, Chinese Academy of Sciences, Ningbo, 315201 People’s Republic of China; 2 School of Chemical Sciences, University of Chinese Academy of Sciences, Beijing, 100049 People’s Republic of China; 3 School of Materials Science and Chemical Engineering, Ningbo University, Ningbo, 315211 People’s Republic of China

**Keywords:** Stimuli-responsive hydrogels, Touch stimulation, Bionic actuation, Smart materials, Supersaturated salt solution

## Abstract

Stimuli-responsive hydrogel is regarded as one of the most promising smart soft materials for the next-generation advanced technologies and intelligence robots, but the limited variety of stimulus has become a non-negligible issue restricting its further development. Herein, we develop a new stimulus of “touch” (i.e., spatial contact with foreign object) for smart materials and propose a flytrap-inspired touch-responsive polymeric hydrogel based on supersaturated salt solution, exhibiting multiple responsive behaviors in crystallization, heat releasing, and electric signal under touch stimulation. Furthermore, utilizing flytrap-like cascade response strategy, a soft actuator with touch-responsive actuation is fabricated by employing the touch-responsive hydrogel and the thermo-responsive hydrogel. This investigation provides a facile and versatile strategy to design touch-responsive smart materials, enabling a profound potential application in intelligence areas.

**Supplementary Information:**

The online version contains supplementary material available at 10.1007/s40820-022-00931-4.

## Highlights

- A pioneering touch-responsive material with multiple responsiveness to touch stimulation (i.e., spatial contact with foreign object) is successfully developed.
- The responsive behaviors of touch-responsive hydrogel exhibit excellent controllability and designability.
- Based on cascade response strategy, a flytrap-like soft actuator with touch-responsive actuation is fabricated by employing the touch-responsive hydrogel.

**Supplementary Information:**

The online version contains supplementary material available at 10.1007/s40820-022-00931-4.

## Graphical abstract

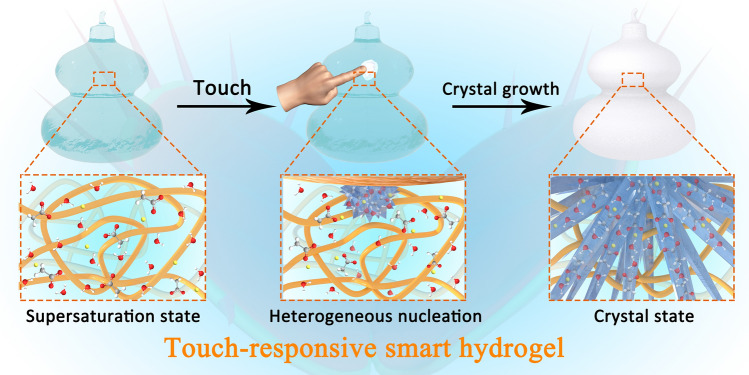

## Introduction

Taking inspiration from smart biological tissues, artificial soft actuators fabricated by stimuli-responsive soft materials offer fascinating prospects owing to their remarkable advantages in compliance, safety and degree of freedom, benefiting to meet the growing need for soft robots [1–4]. Bearing great similarities to biological systems, hydrogels with soft and wet feature have attracted tremendous interest in the field of soft actuators [5–7]. The structural feature of three-dimensional polymer network containing a large amount of water enables reversibly swelling and shrinking by absorbing or exuding water for hydrogels in response to external stimulus, this performance provide a base for the actuations of most hydrogel-based actuators. Therefore, developing the stimuli responsiveness of hydrogels is critical to promoting the progress of hydrogel-based actuators.

Stimuli-responsive hydrogels, which are capable of changing their physicochemical properties in response to the specific external stimulus, are deemed to be one of the most important building blocks of the next-generation advanced technologies and intelligence devices [8–10]. Over the past decade of booming development, various kinds of smart hydrogels with stimuli responsiveness have been developed by modifying stimuli-responsive polymers [11–13], introducing functional additives [14–16] and designing ingenious structures [17–19]. At this stage, the stimuli-responsive smart hydrogels show multi-type changes in size, shape, optical properties, mechanical properties and electric properties adapting to various external stimuli [20–27], and they have been widely applied in soft actuators [5, 28–30] and other intelligence areas [31–38]. For example, Wu's group [39–41] reported a series of smart hydrogels with elaborately ordered structures of nano-additives, which capable of programmed deformations and actuations as soft robots under stimulation of heat, light, and electric field. Recently, Sun et al. [42] proposed a novel electroosmotic turgor actuator by confining smart hydrogel with a selectively permeable membrane, and this responsive hydrogel exhibited substantially faster and stronger responding actuation compared other osmotic hydrogels. Although a great deal of efforts have been taken to improve the response performance of smart hydrogels and actuators, and many breakthroughs have been gained, there is still a huge gap between hydrogel-based actuators and organisms in the stimuli-responsive actuation process due to the limitation of the existing stimulation types. Organisms have marvelous irritability to accommodate complicated and changeable environment stimuli, including the touch from foreign object. For example, flytrap can make a rapid responsiveness of shutting snaps when its tiny hairs are touched in spatial by objects [43, 44]. However, this touch stimulation-actuation responsiveness capability is difficult to achieve for hydrogel-based actuators due to the lack of touch-responsive smart hydrogel. Therefore, more biomimetic and efficient stimulation types are desired to be developed for smart hydrogels, enriching the stimuli responsiveness of hydrogels and promoting the progress of intelligent actuators.

If the touch-responsive capability can be developed in hydrogel system, it will not only endow smart hydrogel with responsiveness to new stimulus, but provide a reachable road to fabricate a biomimetic intelligent hydrogel-based actuator with touch stimulation-actuation responsiveness capability. As is known to all, supersaturated solution is metastable, it will rapidly generate crystal nucleus and crystallize when suffering from disturbance [45–49], such as contacting with foreign object. This phenomenon is similar to the touch-responsive performance of flytrap and mimosa. Inspired by this, we proposed a pioneering touch-responsive smart hydrogel via employing the supersaturated sodium acetate (NaAc) solution in hydrogel. Owing to the metastability of supersaturated salt solution, the supersaturated salt endowed the hydrogel with new stimulus—“touch” (i.e., spatial contact with foreign object). This touch-responsive hydrogel exhibited excellent multi-response ability to touch stimulation. More significantly, a flytrap-like hydrogel-based actuator with touch-responsive capacity was developed successfully using biomimetic cascade response strategy, which greatly improve the sensitivity of soft actuator to foreign objects’ touch.

## Experimental Section

### Materials

Acrylamide (AAm), N,N'-Methylenebisacrylamide (MBAA), calcium chloride anhydrous (CaCl_2_), ammonium persulfate ((NH_4_)_2_S_2_O_8_, APS), sodium acetate (NaAc), 2,2-Diethoxyacetophenone (DEAP) were purchased from Aladdin Industrial Co., and sodium acetate trihydrate (CH_3_COONa·3H_2_O) was purchased from Sinopharm Chemical Reagent Co., Ltd.

### Preparation of the Touch-responsive Smart Hydrogel

Typically, the touch-responsive smart hydrogel was prepared by the following steps: Firstly, 1 g sodium acetate trihydrate was molten at 60 °C, and 150 mg AAm and 1.5 mg MBAA were dissolved in the molten solution with continuous stirring. Then, 5 mg APS was dissolved uniformly in the above solution and transferred into mold rapidly before polymerization. Finally, a hydrogel with high-content of sodium acetate was polymerized by thermal-initiation polymerization at 70 °C for 5 min, and the touch-responsive smart hydrogel was obtained when the hydrogel was cooled to room temperature.

### Preparation of the Patterned Touch-Responsive Smart Hydrogel

The touch-responsive smart hydrogel with 0.5-mm thickness was placed on a heating platform of 60 °C, and a PET mask with special pattern was placed on the touch-responsive smart hydrogel. Then, adding a certain amount of deionized water to the touch-responsive smart hydrogel by squeezing a watery sponge, ensuring the exposed surface of the touch-responsive smart hydrogel was covered with deionized water. After standing for 2 min, the excess water and the PET mask were removed from the touch-responsive smart hydrogel, and the patterned touch-responsive smart hydrogel was obtained after cooling.

### Preparation of the CaCl_2_ Crystal Hydrogel

Typically, the CaCl_2_ crystal hydrogel was prepared by the following steps: Firstly, 500 mg CaCl_2_, 150 mg AAm, 1.5 mg MBAA and 5 μL DEAP were dissolved in 500-μL deionized water with continuous stirring at 80 °C. Finally, the CaCl_2_ crystal hydrogel was obtained by placing the precursor solution under UV irradiation (50 W, 365 nm) at room temperature for 3 min.

### Fabrication of the Touch-responsive Soft Actuator

The touch-responsive soft actuator was fabricated by the following steps: Firstly, a CaCl_2_ crystal hydrogel (original size is 9 mm × 6 mm × 0.5 mm) was stretched with a strain of 300%, and then adhering to a pan paper (30 mm × 8 mm × 0.02 mm) based on its stickiness, the pre-stretched state of the CaCl_2_ crystal hydrogel was fixed using glass sheet and two clamps. Subsequently, a touch-responsive smart hydrogel (18 mm × 6 mm × 1 mm) was prepared in situ on the pan paper. Finally, the touch-responsive soft actuator was cooled in a refrigerator of 0 °C, and the CaCl_2_ crystal hydrogel transformed from soft state to stiff state, and the touch-responsive smart hydrogel maintained in supercooling state.

### Thermal Analysis of Touch-responsive Smart Hydrogel

The sample of the touch-responsive smart hydrogel was sealed in an aluminum hermetic pan, then measured by differential thermal analyzer (DSC 214, NETZSCH, Germany) in the temperature range of − 100 to 80 °C. The sample was heated to 80 °C at a heating rate of 5 °C min^−1^ and then held at 80 °C for 10 min. Then, the sample was cooled to − 100 °C at a cooling rate of 5 °C min^−1^ and held for 10 min. Subsequently, the sample was heated to 80 °C at 5 °C min^−1^. Finally, the sample was cooled to 20 °C at 10 °C min^−1^.

### XRD Measurement

The touch-responsive smart hydrogel with supercooling state or crystal state were prepared with film shape, and they were measured by an X-ray powder diffractometer (XRD, D8 Advance DaVinci, Bruker, Germany) using Cu tube with 1.5418 Å.

## Results and Discussion

### Touch-responsive Crystallization and Mechanism of Touch-responsive Hydrogel

**Fig. 1:**
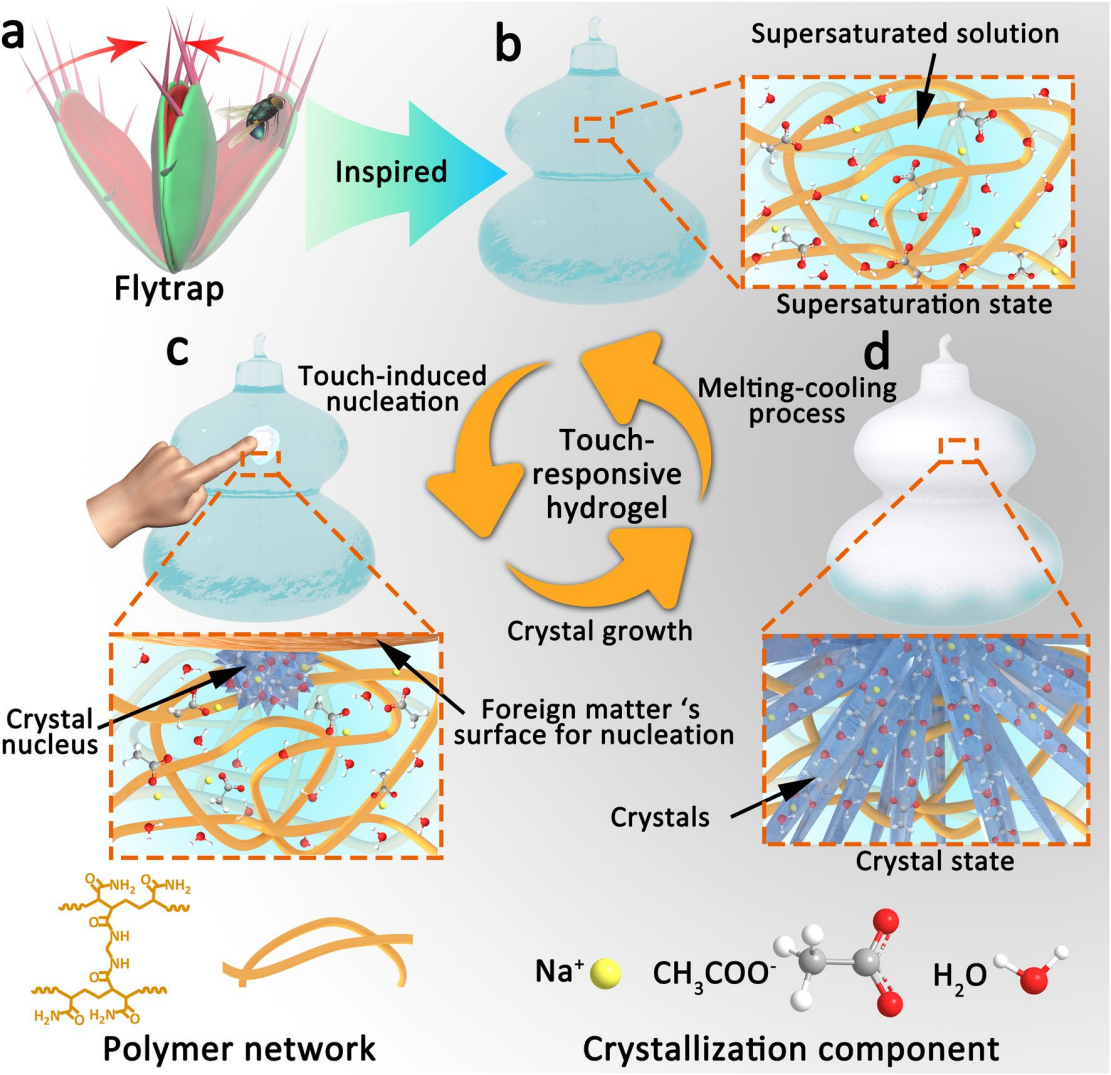
Schematic diagram of the touch-responsive smart hydrogel. **a** Scheme of the flytrap with touch-responsive ability. **b-d** Structure of the touch-responsive hydrogel with **b** supersaturation state, **c** heterogeneous nucleation under touch stimulation, **d** crystal state

In this study, the touch-responsive hydrogel was prepared through the following steps. First, the precursor solution was prepared by mixing monomer (acrylamide), crosslinker (N,N'-Methylenebisacrylamide) with molten sodium acetate trihydrate at 60 °C, where the weight percentage of monomer relative to salt hydrate is 15 wt%. Then, the thermal initiator (ammonium persulfate) was added to the precursor solution and dispersed rapidly before polymerization. Finally, a transparent hydrogel was formed at 70 °C for 5 min, and the touch-responsive smart hydrogel contains supersaturated salt solution was obtained successfully when the polymerized hydrogel was slowly cooled to room temperature. Due to the high energy barrier, it is difficult for supersaturated salt solution to nucleation, thus it can remain stable within polyacrylamide matrix. Furthermore, the hydrogen interaction between polyacrylamide networks and sodium acetate also suppresses the molecular integration, and improves the stability of the metastable supersaturated salt solution to some extent (Fig. 1b). Interestingly, when the touch-responsive hydrogel with supersaturation state was gently touched by foreign object, the supersaturated NaAc solution can form crystal nucleus instantaneously at the point of contact (Fig. 1c), and then the NaAc crystals grow throughout the entire hydrogel rapidly (Fig. 1d). Of course, the crystallized hydrogel can transform to supersaturation sate again after heating and natural cooling process, realizing the regeneration of touch-responsive smart hydrogel.

**Fig. 2:**
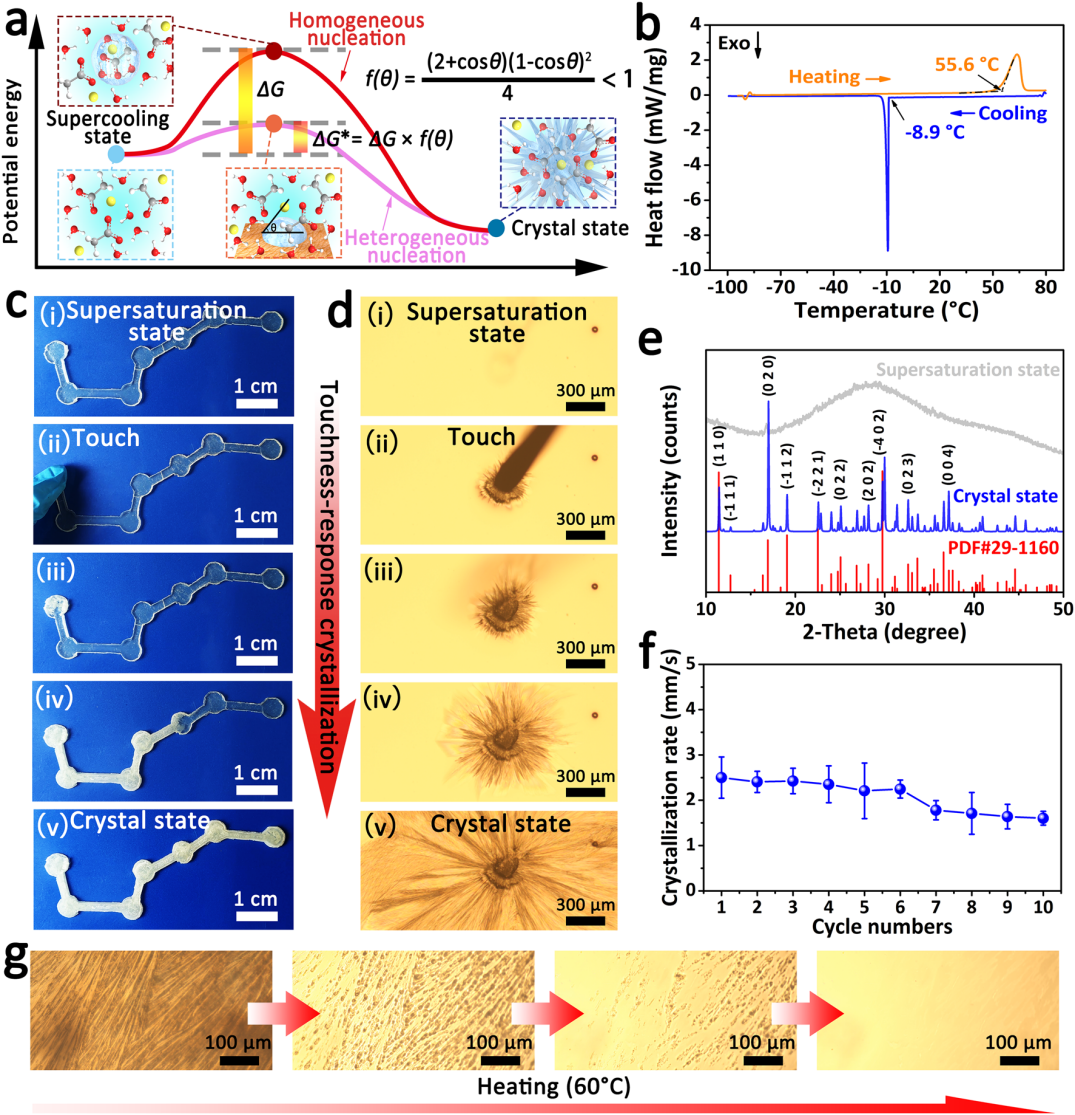
Crystallization and supersaturation behaviors of the touch-responsive hydrogel. **a** Schematic diagram of nucleation energy barrier of homogeneous nucleation (*∆G*) and heterogeneous nucleation (∆G*). **b** DSC of the touch-responsive hydrogel in the range of -100 to 80 °C, at heating and cooling rate of 5 °C min ^−1^. **c** Photographs showing the touch-responsive crystallization process of the touch-responsive hydrogel. **d** Touch-responsive crystallization process of the touch-responsive hydrogel by polarization microscope. **e** XRD spectrums of the touch-responsive hydrogel in different states, and the red spectrum is the standard spectrum of sodium acetate trihydrate. **f** Crystallization rates of the touch-responsive hydrogel in the ten crystallization melting cycles. **g** Polarization microscope images showing the melting process of the touch-responsive hydrogel on 60 °C heating platform

The touch-responsive capability of our hydrogel mainly attributes to the transformation of nucleation type caused by touch. Within the framework of classical nucleation theory, crystals are formed from a supersaturated solution via nucleation and subsequent growth. The initial nucleation process, leading to the formation of a new crystalline phase, can be divided into homogeneous nucleation (nucleating in the volume) and heterogeneous nucleation (nucleating on foreign surface) [50]. According to the thermodynamic model of nucleation, heterogeneous nucleation has a lower nucleation energy barrier (between supersaturation state and critical state) compared with homogeneous nucleation [51–53]. As shown in Fig. 2a, the supersaturated salt solution within touch-responsive smart hydrogel is difficult to spontaneously nucleate in the absence of external disturbance due to the high nucleation energy barrier of homogeneous nucleation. But what is really interesting is touching the touch-responsive hydrogel can transform the nucleation type of supersaturated salt solution from homogeneous nucleation to heterogeneous nucleation, whose lower energy barrier enables supersaturated salt solution fast nucleate on the surface of a foreign object and grow in the hydrogel. Meanwhile, the kinetic energy caused by touch will disturb the supersaturated salt solution and accelerate the movement of salt molecules, which destroying the metastable state of the supersaturated salt solution and promoting the formation of crystal nucleus. Therefore, the touch-responsive smart hydrogel is very sensitive to the stimulation of touch from foreign object.

The thermal properties of the touch-responsive smart hydrogel were analyzed by the differential scanning calorimetry (DSC). As shown in Fig. 2b, the solid–liquid transition temperature and liquid–solid transition temperature of the touch-responsive hydrogel were ~ 55.6 and ~ -8.9 °C, respectively. The maximum supercooling temperature reached about ~ 64.5 °C, indicating that the touch-responsive hydrogel can maintain the metastable supersaturation state over a wide temperature range. We observed the touch-responsive crystallization behavior of the touch-responsive smart hydrogel. As shown in Fig. 2c and Video S1, the supersaturated touch-responsive hydrogel was transparent and stable before touch, and a white region occurred at the point of touch when the touch-responsive hydrogel was touched by foreign object because of the formation of crystal nucleus and the generation of NaAc crystals. With the growth of crystals, the white region expanded forward rapidly and eventually spread throughout the entire hydrogel. Similar phenomenon could be observed by polarizing microscope. As the touch-responsive hydrogel was touched by a stick, needle NaAc crystals emerged instantaneously and grew in all direction quickly (Fig. 2d and Video S2). Besides, the crystallization rate of the responsive crystallization was measured under different environmental temperatures. As shown in Fig. S1, the crystallization rate remained about 2.5 mm s^−1^ at low temperature (below 30 °C) under the suppression of polymers. When the temperature is above 30 °C, the crystallization rate decreased with the increasing environmental temperature because the degree of supercooling is the driving force. The fast responsive behavior under touch endowed the touch-responsive smart hydrogel with a potential application in trajectory tracking using the array configuration (Fig. S2 and Video S3). The differences of the touch-responsive hydrogel before and after being touched were analyzed by XRD. As shown in Fig. 2e, the crystallized touch-responsive hydrogel after touch showed obvious diffraction peaks, which is matched with the spectrum of sodium acetate trihydrate crystals (PDF#29-1160), while there are no diffraction peaks for the supersaturated touch-responsive hydrogel. This result proved that the supersaturated solution within hydrogel was induced to crystallize after touch. When the crystallized touch-responsive hydrogel was heated at a high temperature (60 °C, higher than the transition temperature of touch-responsive hydrogel), the needle crystals within hydrogel was molten slowly, and the touch-responsive hydrogel was obtained again after cooling (Fig. 2g and Video S4). Furthermore, although the touch-responsive hydrogel showed a downward trend in crystallization rate during the crystallization-melting cycles due to the water loss in heating process, the stability is satisfactory (Fig. 2f). The stable cycle performance of the touch-responsive hydrogel provides the basis for its application, such as touch-triggered information display (Fig. S3).

**Fig. 3:**
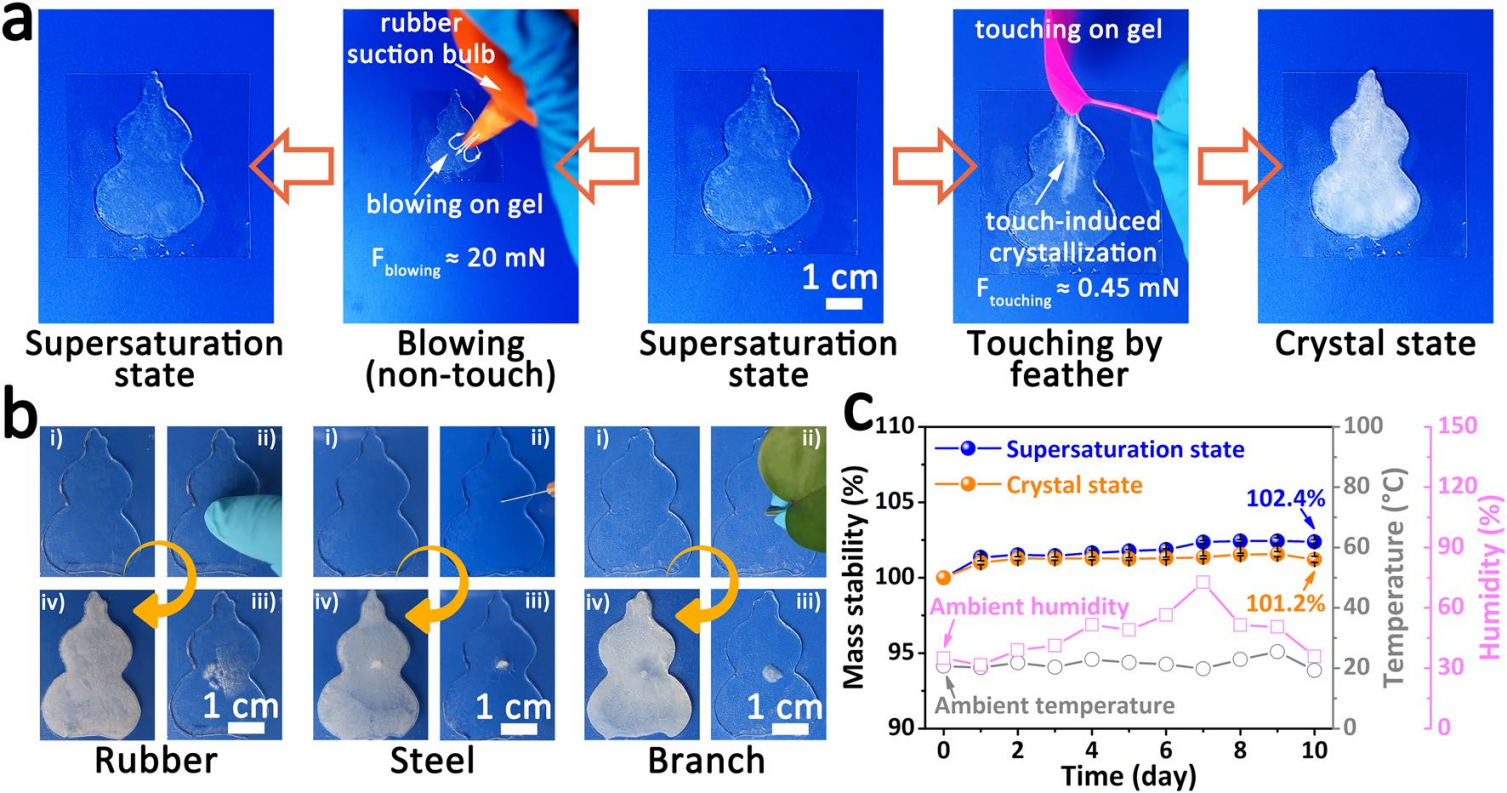
Mechanism validation of the touch-responsive smart hydrogel. **a** Responsive behavior comparison of the touch-responsive hydrogel under different stimulations, including the blowing with big force but non-touch, gently touch with small force. **b** Photographs showing the touch-responsive crystallization processes of the touch-responsive hydrogel under the touch by rubber, steel, and branch. **c** Mass variations of the supersaturated touch-responsive hydrogel and the crystallized touch-responsive hydrogel in an open natural environment

In order to verify the touch-responsive behavior is mainly derived from the spatial contact with foreign object rather than the external force on hydrogel, a comparative experiment was carried out. As shown in Fig. 3a and Video S5, it was found that the supersaturated touch-responsive hydrogel was induced to crystalize when a feather touched the hydrogel lightly with a small force (~ 0.45 mN, calculated by high precision electronic balance), while crystallization never occurred even suffered from a much larger force (~ 20 mN) when the foreign object did not directly contact with hydrogel. Actually, the smart hydrogel could be induced to crystallize even at force as low as ~ 0.1 mN (Fig. S4). This result confirmed that the spatial contact is an important precondition for this touch-responsive behavior, and the inevitable force on hydrogel is not the key driver for this smart process. Based on this mechanism, the touch-responsive smart hydrogel is little selective about the foreign objects, and this touch-responsive crystallization could be induced by most solid objects, such as rubber, steel, branch, glass, plastic, and skin (Fig. 3b and Fig. S5). Of course, the contact force did not affect the crystallization rate significantly because the crystallization rate mainly depends on the degree of supercooling (Fig. S6). Additionally, both the supersaturated touch-responsive hydrogel and the crystallized touch-responsive hydrogel exhibited satisfactory stability in an open natural environment, although there was a slight increment (1 ~ 2 wt%) in weight due to the water absorption of high-content salt system (Fig. 3c).

**Fig. 4:**
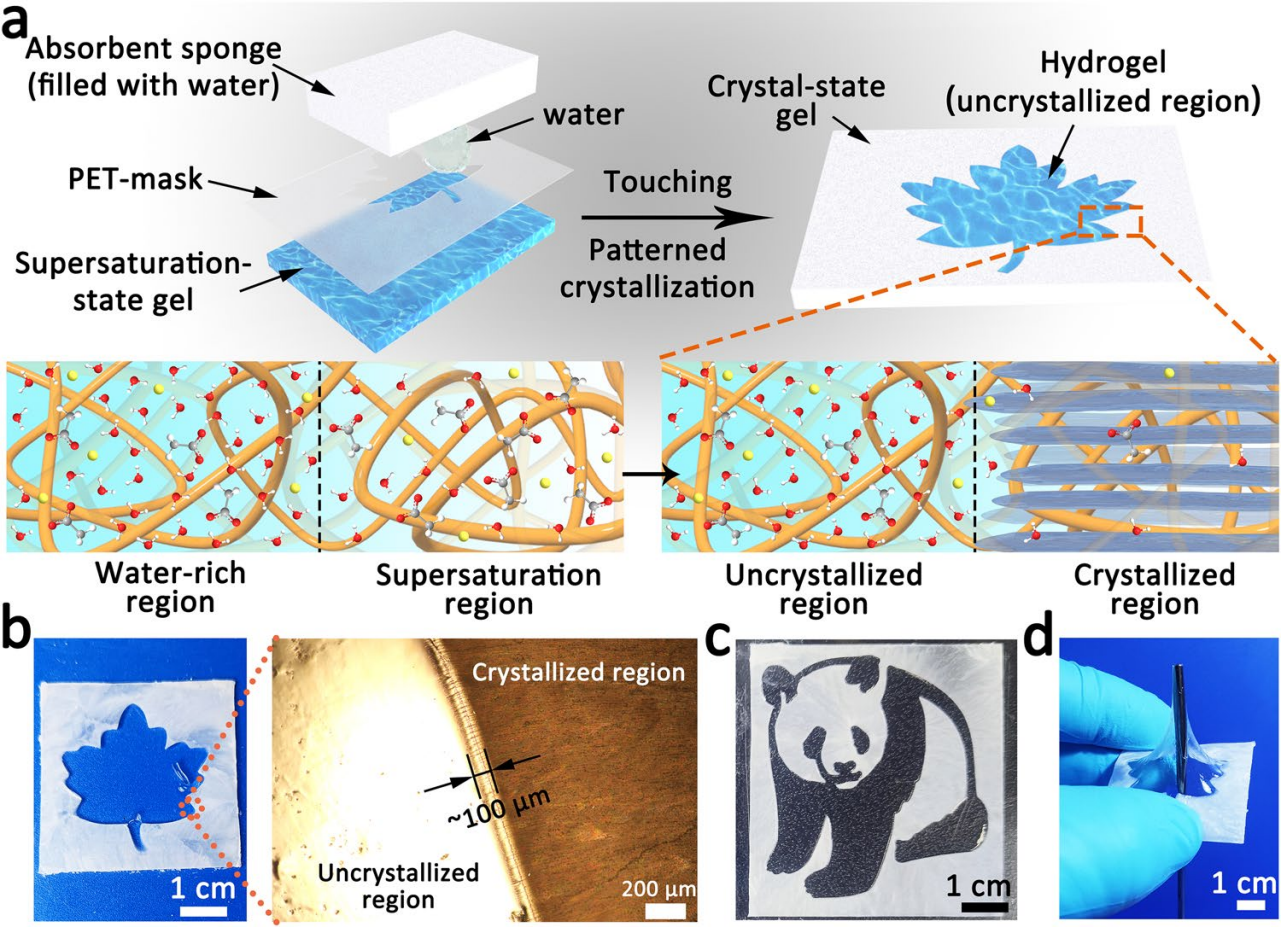
Patterned crystallization of the touch-responsive smart hydrogel. **a** Schematic diagram of the touch-responsive patterned crystallization by adding water in local region with the help of patterned mask. **b** Patterned crystallized touch-responsive hydrogel (thickness is 0.5 mm) with a pattern of “maple leaf.” The right picture is microscopic image of the patterned crystallized touch-responsive hydrogel by polarization microscope. **c** Patterned crystallized touch-responsive hydrogel with a pattern of “panda.” **d** Photograph showing the mechanical properties of the patterned crystallized touch-responsive hydrogel, being punctured by needle

The responsive crystallization behavior of touch-responsive hydrogel exhibits excellent controllability and designability. Supersaturation is the foundation for the touch-responsive smart hydrogel to realize crystallization under touch stimulation; therefore, the crystallization area can be controlled by adjusting the distribution area of the supersaturated salt solution. As shown in Fig. 4a, a touch-responsive hydrogel (0.5 mm in thickness) containing local supersaturation region can be prepared with the assistance of PET mask and watery absorbent sponge, and the patterned crystallization response can be realized under touch stimulation. By squeezing the sponge, deionized water was added into touch-responsive hydrogel’s supersaturation region through the mask, and the supersaturation region transforms to water-rich unsaturation region, where cannot be induced to crystallize. Utilizing this strategy, a crystallized hydrogel with leaf-pattern uncrystallized region was obtained rapidly after touching the supersaturation region (Fig. 4b and Video S6). It is worth noting that the size of the transition zone between crystallized region and uncrystallized region is about 100 μm, revealing that this patterned crystallization method possesses a high accuracy. As shown in Figs. 4c and S7, various elaborate patterns could be readily displayed by adding water onto the designed masks and touching the patterned touch-responsive hydrogel. Unfortunately, the pattern stability still needs to be improved because of the diffusion of water molecular (Fig. S8). Besides, due to the significant difference in mechanical properties of two regions (Figs. 4d and S9), this touch-responsive patterned crystallization provide an efficient method to spatially modulate the stiffness of soft hydrogel and design rigid-soft coupling materials.

### Exothermal and Electric Behaviors of Touch-responsive Smart Hydrogel

**Fig. 5:**
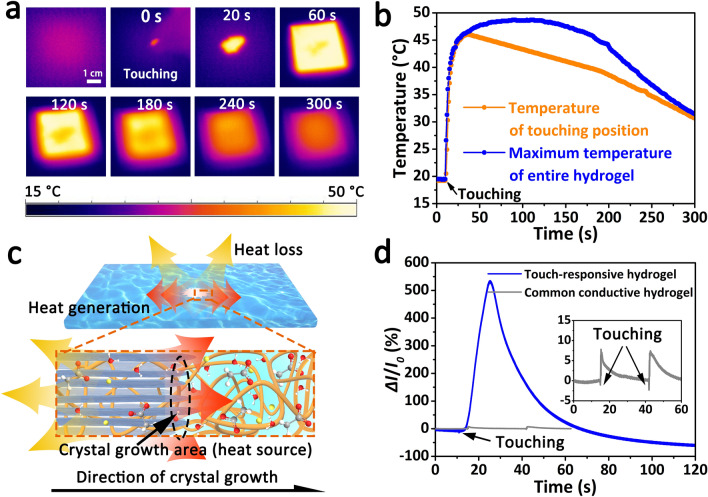
Multi-responsiveness of the touch-responsive smart hydrogel under touch stimulation. **a** Time-dependent infrared thermal images of the touch-responsive hydrogel under touch stimulation. **b** Temperature variation curves of the touch-responsive hydrogel under touch stimulation, including the temperature of touching position and the maximum temperature of entire hydrogel. **c** Responsive exothermic schematic diagram of the touch-responsive hydrogel during the crystallization. **d** Relative electric current variation of the touch-responsive hydrogel and the common conductive polyacrylamide hydrogel with 1 M sodium acetate under touch stimulation

In addition to the responsive crystallization, exothermal behavior is also an important responsiveness for the touch-responsive hydrogel under touch stimulation because the crystallization of NaAc solution is accompanied by the release of latent heat. The dynamic temperature maps of the touch-responsive hydrogel during the touch-responsive process were recorded by an infrared camera, and its temperature changes in spatial and temporal resolution were investigated (Fig. 5a). As shown in Fig. 5b, when the touch-responsive hydrogel was touched, the temperature of touching position rapidly increased from ambient temperature (20 °C) to ~ 46 °C in 20 s, and then dropped slowly as the dissipation of heat. Interestingly, the maximum temperature of the entire hydrogel is slightly higher than the maximum temperature of touching position, and lasts longer. This is because the latent heat of the supersaturated salt solution is released (heat generation) predominantly at the crystallization front and subsequently dissipates (heat loss) by diffusive heat transfer into the surrounding environment (including surrounding hydrogel and air) during crystal growth (Fig. 5c). For a certain position, its temperature increased when the obtained heat (including the released latent heat and the received heat transferred from surrounding hydrogel) is greater than the dissipated heat, otherwise decreased. Therefore, the maximum temperature of the entire touch-responsive hydrogel moves forward along the crystal growth synchronously.

More significantly, a remarkable change in the electric signal could be observed when the touch-responsive hydrogel was touched, which is far greater than the common conductive hydrogel (Fig. 5d). This is explained by the fact that the increased temperature caused by touch can greatly improve the ionic conductivity of the touch-responsive hydrogel at the beginning, leading to an amazing improvement (> 500%) in electric current within a short time. Subsequently, the number of free ions (Na^+^, CH_3_COO^−^) gradually reduces due to crystallization, resulting in a decreasing electric current. This responses in electric signal under touch stimulation provides a potential application in detecting the touch behavior from foreign object.

### Flytrap-like Touch-Responsive Soft Actuator

**Fig. 6:**
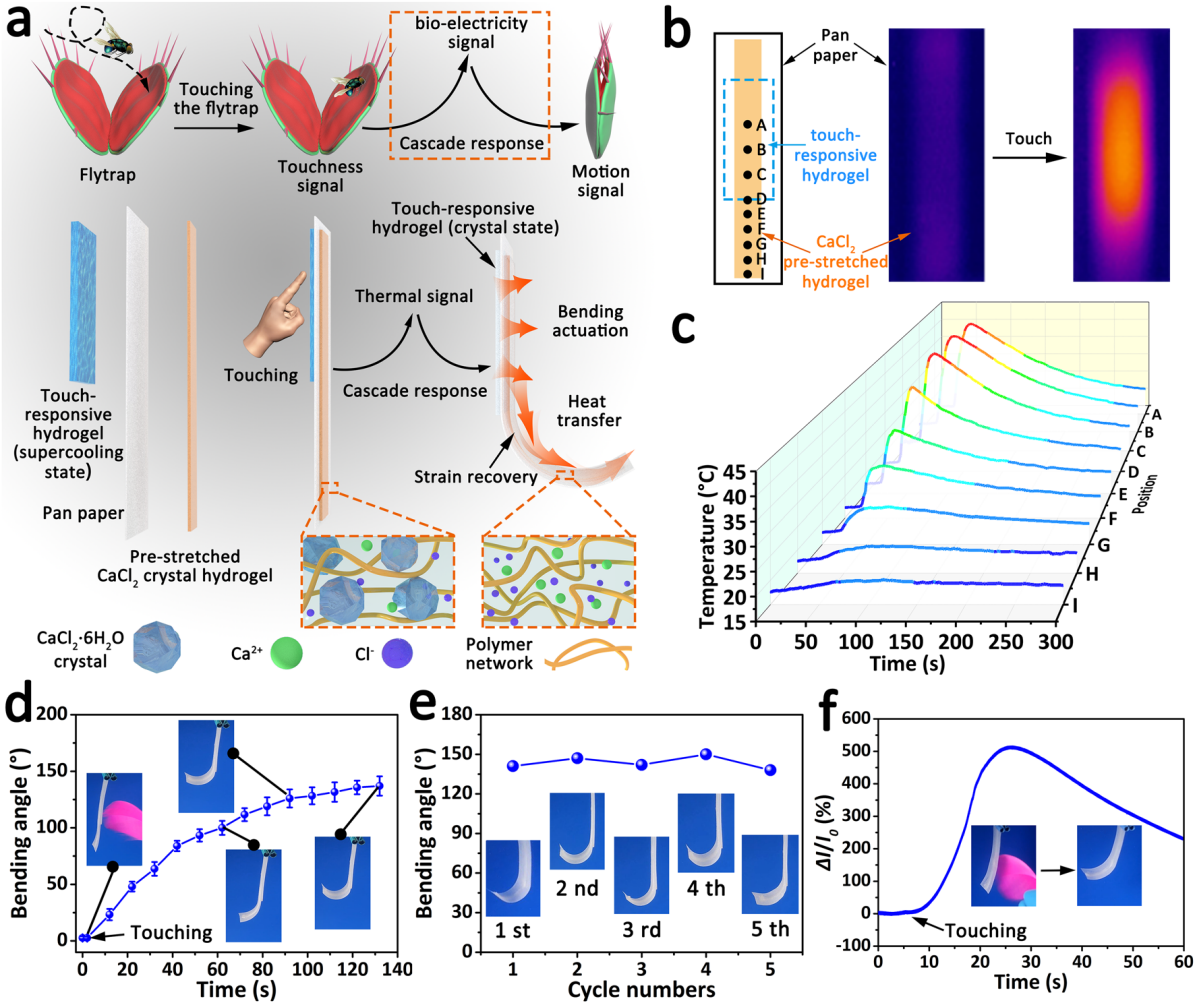
Fabrication and characterization of touch-responsive soft actuator. **a** Schematic diagram of the flytrap-like touch-responsive soft actuator based on the touch-responsive smart hydrogel. **b** Structure of the touch-responsive soft actuator (left) and the infrared thermal images of the pre-stretched CaCl_2_ crystal hydrogel before (middle) and after touching (right). **c** Temperature variation curves of the pre-stretched CaCl_2_ crystal hydrogel at different positions during the responsive crystallization process. The space of position A ~ D is 4 mm, and the space of position D ~ I is 2 mm. **d** Bending angle variation of the touch-responsive soft actuator after touch. **e** Stability of the touch-responsive soft actuator in the cyclic bending actuation. **f** Relative electric current variation of the touch-responsive soft actuator under touch stimulation

Flytrap is a typical touch-responsive plant, who can generate bio-electric signal response to the touch stimulation from prey, and then drive its trap to close in response to the bio-electricity signal. Inspired to this cascade response process, a touch-responsive soft actuator based on a cascade transition of “touch stimulation-thermal signal-actuation responsiveness” was fabricated by using supersaturated touch-responsive smart hydrogel, pan paper and pre-stretched CaCl_2_ crystal hydrogel (Fig. 6a). The CaCl_2_ crystal hydrogel was prepared by one-step polymerization of acrylamide in the high-content CaCl_2_ solution (50 wt%). This CaCl_2_ crystal hydrogel possessed a reversible soft-to-hard transition property (Fig. S10a-b) because of its phase transition at 18.8 °C (Fig. S10c). Besides, the soft CaCl_2_ crystal hydrogel exhibited good elasticity when it was stretched (Fig. S10d). Utilizing the soft-to-hard transition of the CaCl_2_ crystal hydrogel, the touch-responsive soft actuator was fixed through pre-stretching the CaCl_2_ crystal hydrogel with 300% strain at room temperature and subsequently cooling the actuator (attached by the pre-stretched CaCl_2_ crystal hydrogel) at 0 °C. The stimuli-responsive process of the soft actuator consists of the following steps. Firstly, a lot of heat is generated when the touch-responsive hydrogel is touched due to the touch-induced exothermal crystallization. Then, the heat is transferred to the fixed pre-stretched CaCl_2_ crystal hydrogel through heat conduction and its temperature increased gradually. Finally, the pre-stretched CaCl_2_ crystal hydrogel tends to recover to its original length when its temperature exceeds the soft-to-hard transition temperature (18.8 °C), and the soft actuator is driven to bend.

The actuator’s temperature changes with spatial and temporal resolution were measured by an infrared camera. As shown in Fig. 6b, c, the temperature at different positions of the soft actuator increased to varying degrees. Although position showed a lower maximum temperature farther away from the touch-responsive hydrogel, the maximum temperature of position “I” (10 mm from the edge of the touch-responsive hydrogel) was still higher than 20 °C, which ensures that the phase transitions can happen. As shown in Fig. 6d and Video S7, the bending angle of the soft actuator reached about 137° in 130 s after touching, revealing excellent responsive bend capacity under touch stimulation. Besides, the soft actuator exhibited relatively good stability in the early touching stimuli-bending response cycles (Fig. 6e). Of course, there is an inevitable decline in bending angle during the long-term cycles due to the decreasing crystallization rate and maximum temperature of hydrogel (Fig. S11). Of greater significance, a significant change in current signal could be detected when the touch-responsive soft actuator was driven by touch stimulation due to the responsive electric property of touch-responsive hydrogel (Fig. 6f). This multi-responses of touch-responsive soft actuator in bending actuation and electric signal offer bright prospects for its potential application in self-sensing smart actuators and robots.

## Conclusions

In summary, a stimuli-responsive smart hydrogel with biomimetic touch-responsive ability was strategically developed through the polymerization of compatible monomer in molten salt hydrate at high temperature and the subsequent natural cooling process. Utilizing the metastability of the supersaturated NaAc solution within the polymer matrix, there is a startling responsive crystallization under touch stimulation owing to the lower nucleation energy barrier of heterogeneous nucleation on a foreign surface. In addition to the responsive crystallization, the touch-responsive smart hydrogel also possessed excellent responsive exothermal behavior and responsive electric signal due to the simultaneous changes in physicochemical property accompanied by crystallization. More significantly, based on the cascade transition of “touch stimulation-thermal signal-actuation responsiveness,” a flytrap-like touch-responsive soft actuator employed with touch-responsive hydrogel and pre-stretched phase transition hydrogel exhibited satisfactory actuating capacity after being touched by foreign object. Therefore, this promising touch-responsive smart hydrogel based on supersaturated salt solution not only provides a new stimulus for smart materials, but also endows stimuli-responsive hydrogel with diversified responsiveness, enabling a profound potential application in the next-generation intelligence technologies and devices, such as intrusion detector, trajectory tracking system and self-sensing soft actuators/robots.

## Supplementary Information

Below is the link to the electronic supplementary material.

Supplementary file1 (PDF 1534 KB)

Supplementary file2 (MP4 13920 KB)

Supplementary file3 (MP4 14325 KB)

Supplementary file4 (MP4 10572 KB)

Supplementary file5 (MP4 13199 KB)

Supplementary file6 (MP4 9852 KB)

Supplementary file7 (MP4 8453 KB)

Supplementary file8 (MP4 10054 KB)
