# Supplementary material for: Touch-Responsive Hydrogel for Biomimetic Flytrap-Like Soft Actuator
Source: Nanomicro Lett. 2022 Sep 5;14:182. doi: 10.1007/s40820-022-00931-4 (PMC9445118; doi:10.1007/s40820-022-00931-4)
Supplement: Supplementary file 1 — Supplementary file1 (PDF 1534 KB) [file 40820_2022_931_MOESM1_ESM.pdf]

Supporting Information for

Touch-Responsive Hydrogel for Biomimetic Flytrap-Like Soft Actuator

Junjie Wei[1, 2, *], Rui Li[1, 2, 3], Long Li[1, 2, 3], Wenqin Wang[3], Tao Chen[1, 2, *]

[1]Key Laboratory of Marine Materials and Related Technologies, Zhejiang Key Laboratory of Marine Materials and Protective Technologies, Ningbo Institute of Materials Technology and Engineering, Chinese Academy of Sciences, Ningbo 315201, P. R. China

[2]School of Chemical Sciences, University of Chinese Academy of Sciences, Beijing100049, P. R. China

[3]School of Materials Science and Chemical Engineering, Ningbo University, Ningbo 315211, P. R. China

*Corresponding authors. E-mail: weijunjie@nimte.ac.cn (Junjie Wei); tao.chen@nimte.ac.cn (Tao Chen)

S1 Preparation of the Common Conductive Hydrogel

Typically, the common conductive hydrogel was prepared by the following steps: Firstly, 150 mg AAm, 1.5 mg MBAA and 5 μL DEAP were dissolved in 850 μL NaAc solution (concentration is 1 M) with continuous stirring at room temperature. The common conductive hydrogel was obtained by placing the precursor solution under UV irradiation (50 W, 365 nm) at room temperature for 15 min.

S2 Mechanical Measurement

The tensile stress-strain properties of the patterned crystallized touch-responsive smart hydrogel and the $CaCl_2$ crystal hydrogel were measured by an electronic universal testing machine (Z1.0, Zwick, Germany). The strip samples, 2 mm in width and 1 mm in thickness, were carried out at the speed of 50 mm min^{-1}.

S3 Optical Microscope Measurement

Optical microscope measurement was carried out by Polarization microscope (BX51, Olympus, Japan) with a heating platform (mK2000, Instec, America).

S4 Temperature Measurement

The temperature was recorded by the infrared camera (Optris PI, Germany) connecting with an analysis software (Optris PIX Connect).

S5 Sensing Performance Measurement

The Real-time electric current (I) of the touch-responsive smart hydrogel and the common conductive hydrogel were measured by an electrochemical workstation (CHI 660E, CH Instruments Co., Ltd) at a constant voltage of 1 V. The relative electric current ($\Delta I/I_0$) of the touch-responsive smart hydrogel is calculated by the following equation:

$$\Delta I/I_0 = \frac{I - I_0}{I_0} \qquad \text{(S1)}$$

where I_0 is the electric current of the touch-responsive smart hydrogel with supercooling state at room temperature.

The touch-responsive smart hydrogel was touched slightly by a finger during the measurement, but the common conductive hydrogel was pressed forcibly by a finger.

S6 Mass Stability Measurement

The touch-responsive smart hydrogel with supercooling state and the touch-responsive smart hydrogel with crystal state were placed in an open ambient environment. Their weights (m_t), the ambient temperature and the ambient humidity were recorded every day. The mass stability (m/m_0) of sample was calculated by the following equation:

$$m_t/m_0 = \frac{m_t}{m_0} \times 100\% \quad \text{(S2)}$$

where m_0 is the original weight of hydrogel sample.

S7 Supplementary Figures

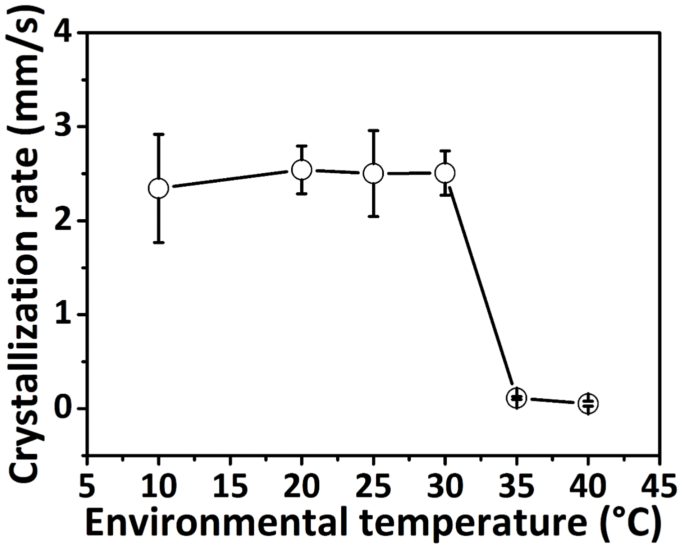

Fig. S1 Crystallization rate of the touch-responsive smart hydrogel under touch stimulation at different environment temperature

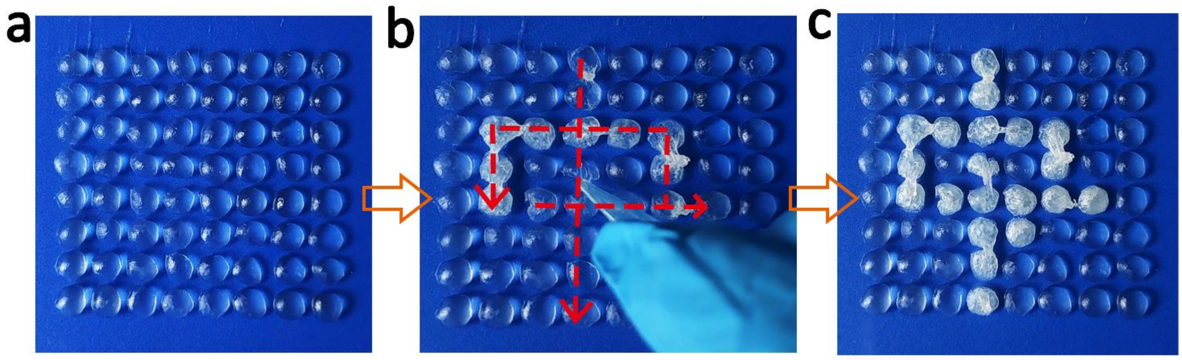

Fig. S2 Demonstration of the touch-responsive smart hydrogel array (8 × 8) applied in trajectory tracking.

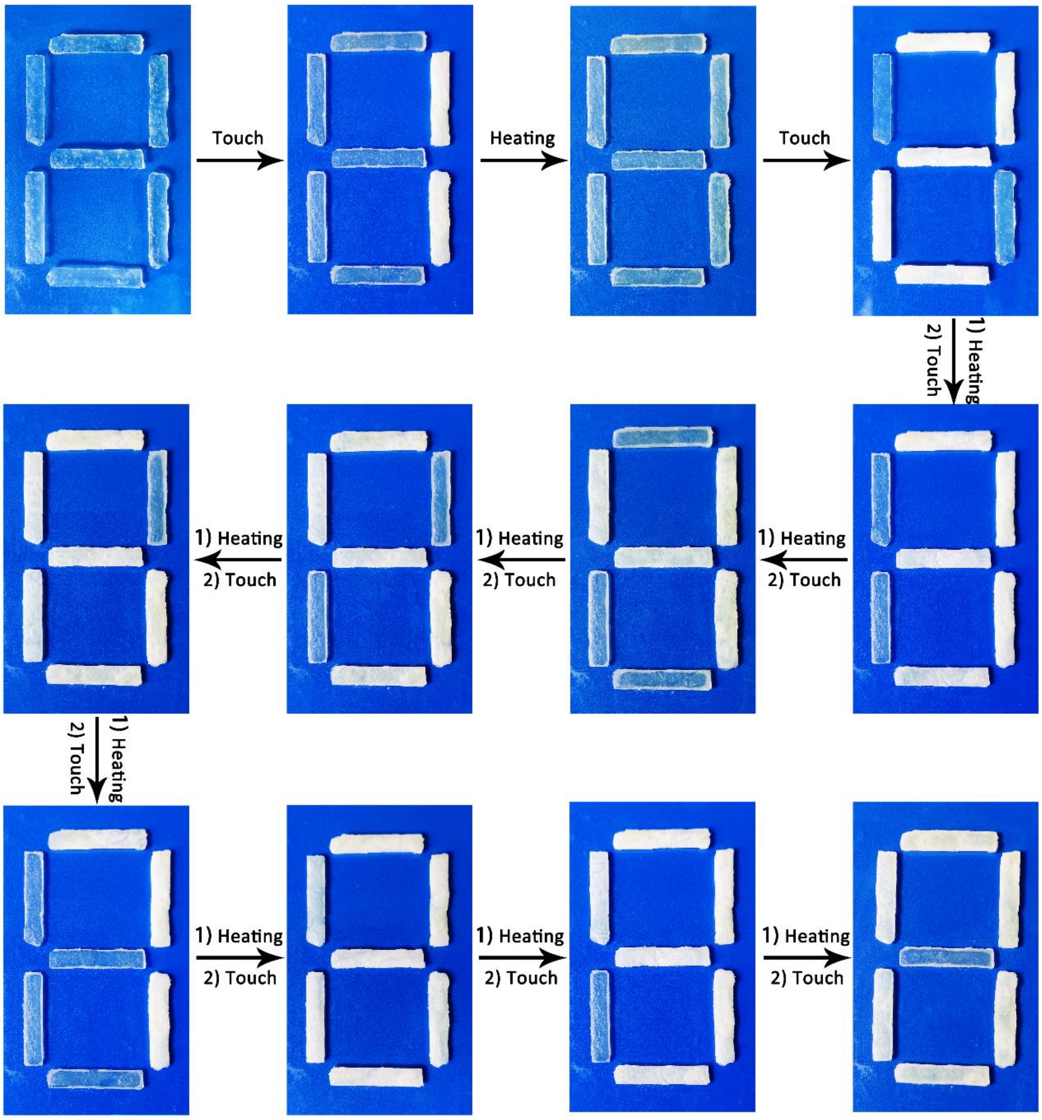

Fig. S3 Erasable information display based on the stability in the heating-crystallization cycles

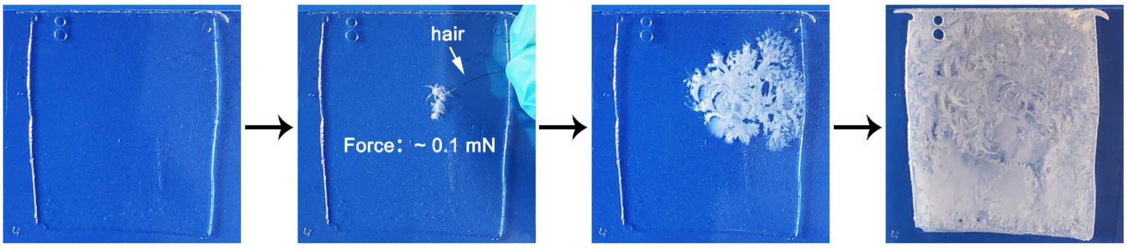

Fig. S4 Responsive crystallization of the touch-responsive smart hydrogel by the touch stimulation from a hair

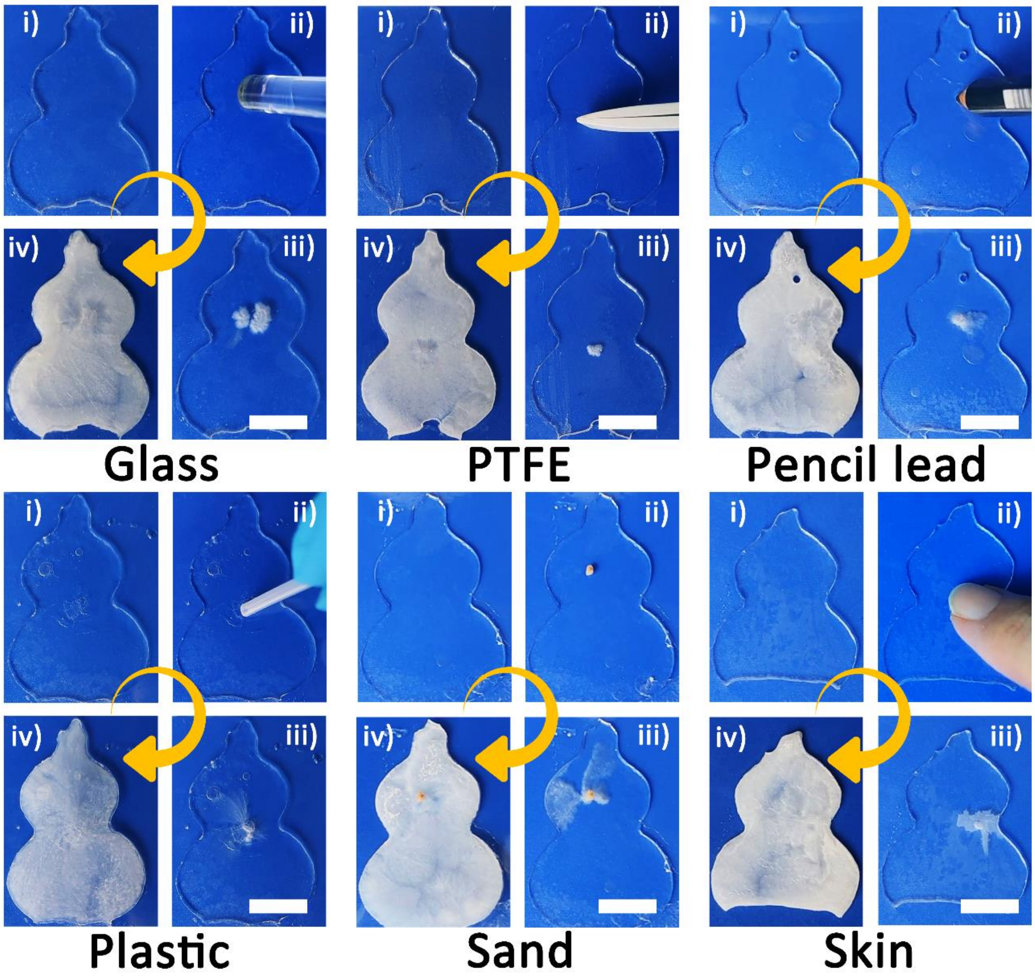

Fig. S5 Photos showing the touch-responsive crystallization processes of the touch-responsive smart hydrogel under the touch by glass, PTFE, Pencile lead, plastic, sand and skin, the scale bar is 1 cm

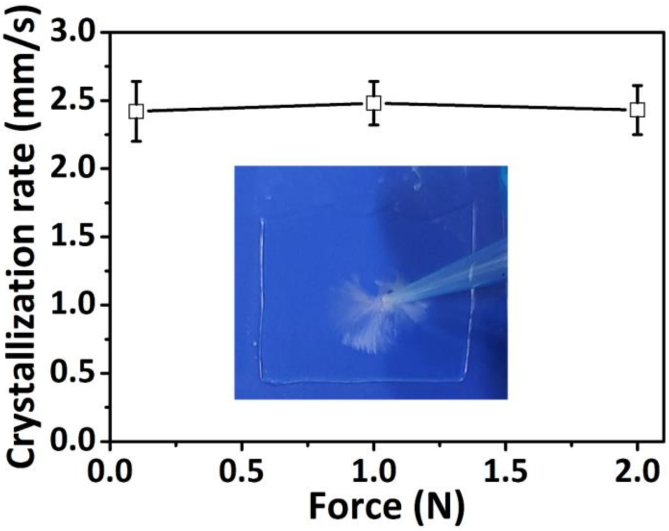

Fig. S6 Effect of Touch force on crystallization rate of the smart hydrogel

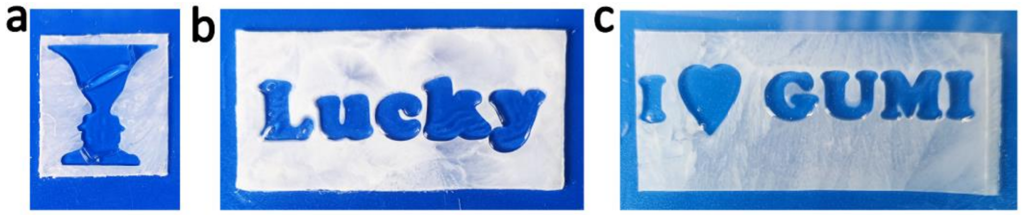

Fig. S7 Patterned crystallized touch-responsive smart hydrogel with various patterns, including (**a**) pattern of "cup", (**b**) English word of "Lucky" and (**c**) the phrase of "I ♡ GUMI"

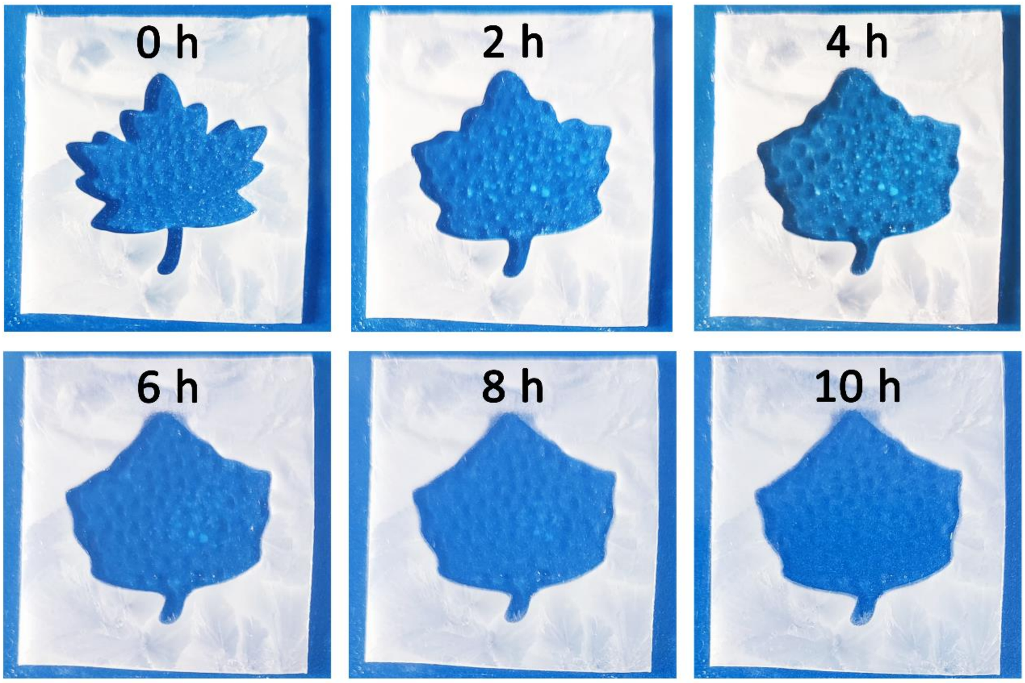

Fig. S8 Long-term stability of the pattern in 10 h

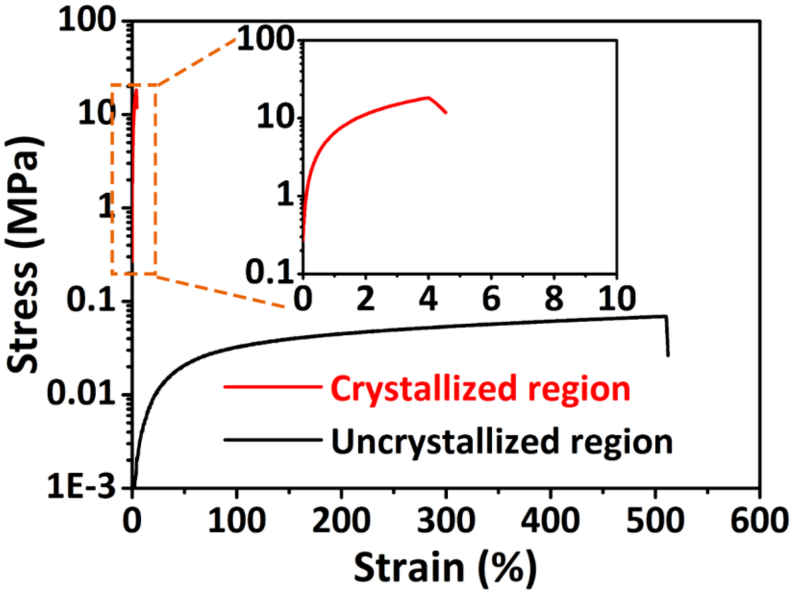

Fig. S9 Tensile curves of the crystallized touch-responsive smart hydrogel and the uncrystallized touch-responsive smart hydrogel at the speed of 50 mm min^{-1}

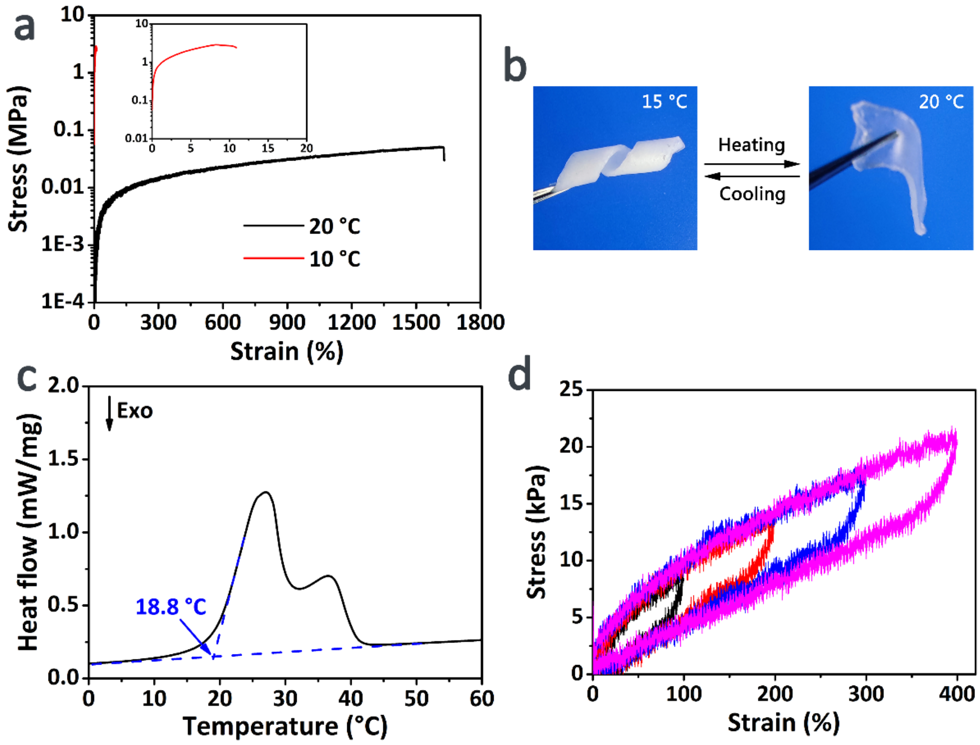

Fig. S10 (**a**) Tensile curves of the $CaCl_2$ crystal hydrogel at temperature of 10 and 20 °C. (**b**) Soft-to-hard transition by adjusting temperature. (**c**) DSC curve of the $CaCl_2$ crystal hydrogel in the range of 0 to 60 °C at heating rate of 5 °C min^{-1}. (**d**) Cyclic tensile curves of the $CaCl_2$ crystal hydrogel with different strain at the speed of 50 mm min^{-1} at 20 °C

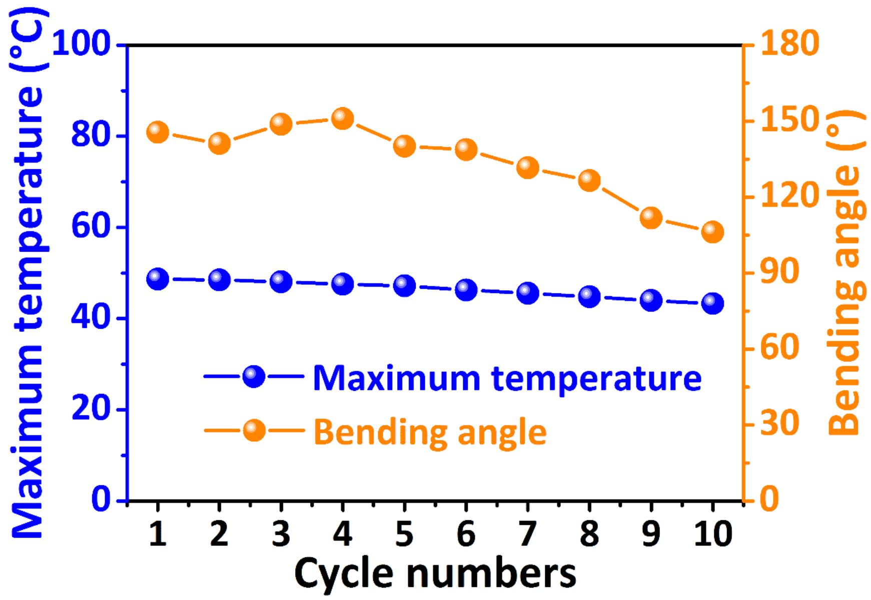

Fig. S11 Stability of the maximum temperature of entire touch-responsive hydrogel and the stability of the bending angle of soft actuator in the 10 cycles
